# Disparities and Temporal Trends in COVID-19 Exposures and Mitigating Behaviors Among Black and Hispanic Adults in an Urban Setting

**DOI:** 10.1001/jamanetworkopen.2021.25187

**Published:** 2021-09-28

**Authors:** Sheila Badri, Vanessa Sardá, Jorge Soria Moncada, Monica Merçon, Katayoun Rezai, Robert A. Weinstein, William E. Trick

**Affiliations:** 1 Department of Medicine, Cook County Health, Chicago, Illinois; 2 Department of Medicine, Rush University Medical Center, Chicago, Illinois; 3 Center for Health Equity and Innovation, Cook County Health, Chicago, Illinois

## Abstract

**Importance:**

COVID-19, caused by SARS-CoV-2 virus, has disproportionately affected Black and Hispanic communities in the US, which can be attributed to social factors including inconsistent public health messaging and suboptimal adoption of prevention efforts.

**Objectives:**

To identify behaviors and evaluate trends in COVID-19–mitigating practices in a predominantly Black and Hispanic population, to identify differences in practices by self-reported ethnicity, and to evaluate whether federal emergency financial assistance was associated with SARS-CoV-2 acquisition.

**Design, Setting, and Participants:**

This survey study was conducted by telephone from July 1 through August 30, 2020, on a random sample of adults who underwent SARS-CoV-2 testing at a safety-net health care system in Chicago during the surge in COVID-19 cases in the spring of 2020. Behaviors and receipt of a stimulus check were compared between participants testing positive and negative for SARS-CoV-2. Differences in behaviors and temporal trends were assessed by race and ethnicity.

**Main Outcomes and Measures:**

SARS-CoV-2 infection was assessed using nasopharyngeal quantitative reverse transcriptase–polymerase chain reaction testing. Mitigating behaviors and federal emergency financial assistance were assessed by survey. Race and ethnicity data were collected from electronic health records.

**Results:**

Of 750 randomly sampled individuals, 314 (41.9%) consented to participate (169 [53.8%] women). Of those, 159 (51%) self-reported as Hispanic and 155 (49%) as non-Hispanic (120 [38.2%] Black), of whom 133 (84%) and 76 (49%) tested positive for SARS-CoV-2, respectively. For all participants, consistent mask use (public transport: adjusted odds ratio [aOR], 0.00; 95% CI, 0.00-0.34; social gatherings: aOR, 0.10; 95% CI, 0.00-0.50; running errands: aOR, 0.18; 95% CI, 0.07-0.42; at work: aOR, 0.23; 95% CI, 0.07-0.79) and hand sanitizer use (aOR, 0.26; 95% CI, 0.13-0.52) were associated with lower odds of infection. During 3 sampled weeks, mitigation practices were less frequent among Hispanic compared with non-Hispanic participants (eg, mask use while running errands: aOR, 0.26; 95% CI, 0.15-0.46). Hispanic participants were at high risk of infection (aOR, 5.52; 95% CI, 4.30-7.08) and more likely to work outside the home (aOR, 2.05; 95% CI, 1.27-3.30) compared with non-Hispanic participants, possibly because of limited receipt of stimulus checks (aOR, 0.03; 95% CI, 0.02-0.07) or unemployment benefits (aOR, 0.36; 95% CI, 0.16-0.74).

**Conclusions and Relevance:**

In this survey study of adults in a large US city, public health messaging improved preventive behaviors over time but lagged among Hispanic participants; messaging tailored to Hispanic communities, especially for mask use, should be prioritized. Hispanic individuals were at higher risk for infection, more often worked outside the home, and were less likely to have received a stimulus check; this suggests larger studies are needed to evaluate the provision of economic support on SARS-CoV-2 transmission dynamics in low-income populations.

## Key Points

**Question:**

What behaviors and disparities in health resources are associated with the spread of COVID-19 in predominantly Black and Hispanic communities?

**Findings:**

In this survey study of adults living in a large US city, consistent masking was associated with a decrease in SARS-CoV-2 acquisition; however, Hispanic individuals were at higher risk for infection, more often worked outside the home, and were less likely to have received economic aid through stimulus checks or unemployment benefits.

**Meaning:**

These results suggest public health messaging may have improved preventive behaviors over time but should be customized for Hispanic communities.

## Teaser

This survey study examines associations of mitigating behaviors and receipt of economic aid with SARS-CoV-2 infection among a group of predominately Black and Hispanic adults tested for SARS-CoV-2 during the first surge of the COVID-19 pandemic in spring 2020.

## Introduction

Coronavirus disease 2019 (COVID-19), caused by the SARS-CoV-2 virus, has disproportionately affected racial and ethnic minority populations in the US.^1,2^ In Chicago, Illinois, Black and Hispanic persons have accounted for 39% and 33% of deaths since the start of the pandemic, respectively, although each represents only 29% of the population.^3^ The disproportionate burden of disease has been attributed to higher rates of transmission and underlying comorbidities associated with worse outcomes.^4,5^ The underlying factors driving spread and severe disease are rooted in adverse social and economic conditions that negatively affect the health of communities, particularly during an infectious disease pandemic.^4,5,6,7,8^ In a prior analysis, we found that ecologic-level neighborhood characteristics were also associated with COVID-19 outcomes.^9^

Prevention efforts such as shelter-in-place, mask use, and physical distancing have been shown to mitigate the spread of SARS-CoV-2.^10,11^ Implementation of these measures in the US has been fragmented and fraught with inconsistent messaging and uptake.^12^ Furthermore, a disproportionate number of Black and Hispanic individuals are employed as essential workers without the capacity to shelter in place or telework.^13^ These complex social and economic dynamics may explain the disparate number of COVID-19 cases in these communities. In an effort to counter the economic effects of COVID-19, Congress approved emergency financial assistance to qualifying US citizens under the Coronavirus Aid, Recovery, and Economic Security (CARES) Act in late March 2020.^14^ Although the goal was to provide economic relief to individuals with reduced income, whether financial assistance was associated with SARS-CoV-2 transmission dynamics has not been evaluated.

In this study, we sought to identify individual behaviors hypothesized to contribute to or mitigate the spread of COVID-19 in predominantly Black and Hispanic populations residing in Cook County, Illinois, during the spring surge of 2020. We also evaluated the association of receiving financial safety-net assistance with the likelihood of SARS-CoV-2 positivity. Lastly, to understand the effectiveness of public health messaging during the early months of the pandemic and to inform future interventions to reduce transmission of SARS-CoV-2 in these populations, we analyzed differences in mitigating behaviors between Hispanic and non-Hispanic participants as well as changes in said behaviors across time. Given the identification of SARS-CoV-2 variants with the potential for increased transmissibility and concerns about vaccine uptake, there is an urgent need to improve nonpharmacologic prevention efforts continuously—before, during, and after wide-scale vaccination distribution.^15,16^

## Methods

### Data Collection

Cook County Health (CCH) is the largest safety-net health care system in Chicago, Illinois, serving a predominantly low-income racial and ethnic minority population. CCH detected its first case of COVID-19 infection in mid-March 2020. A rapid and dramatic increase in cases followed, culminating in peak rates of case detection during April and May 2020. Statewide shelter-in-place orders were initiated on March 21, 2020, with a nadir in individual mobility at the end of March 2020.^17^ On April 3, 2020, the Chicago Department of Public Health recommended face coverings in public settings where social distancing measures were difficult to maintain, avoidance of interactions with people who do not live in the household, social distancing when outside of the home, and frequent hand washing in line with CDC guidance. Statewide mask mandates were issued on May 1, 2020.

Based on high rates of SARS-CoV-2 test positivity rates in our health system’s population, we selected 3 discrete calendar weeks (April 6 through April 13, April 27 through May 3, and May 18 through May 25) to evaluate exposures, mitigating behaviors, and temporal trends in behaviors among adults aged 18 years or older, with the goal of analyzing differences by race and ethnicity. We identified patients who underwent nasopharyngeal quantitative reverse transcriptase–polymerase chain reaction (RT-PCR) testing at any CCH clinical location within the system’s integrated electronic health record. We excluded individuals who resided outside of Cook County, lived in congregate settings (eg, jail), were undomiciled at the time of testing, or were known to have died at the time of data collection. Then we used simple randomization to select 250 adults from each of the chosen calendar weeks with a ratio of participants testing SARS-CoV-2 positive (ie, the case group) to SARS-CoV-2 negative (the control group) of 2:1; our final sample was 500 cases and 250 controls.

Study personnel contacted potential participants by telephone for a wellness check call since testing for COVID-19 and were then invited to participate in a structured survey by verbal consent. Three phone calls per participant were attempted, and efforts were made to schedule the survey call at participants’ convenience. The survey was developed by the study team through an iterative review process. Spanish translation was performed by research personnel with experience in English-to-Spanish translation. The survey translation was pilot tested on native Spanish speakers to assess for language validity and cross-cultural understanding. Surveys were conducted in English or Spanish depending on patient preference over 8 weeks from July 1 to August 30, 2020. Demographic data (ie, age, gender, self-reported race and ethnicity) of respondents were collected from the electronic health record.^18^ All survey data were collected and managed using REDCap software version 11.1.2 hosted at CCH.^19^ Participants were offered a $20 gift card in compensation for their time. The CCH institutional review board reviewed and approved the study with a waiver of informed consent as the study did not involve more than minimal risk to participants.

### Survey

The 36-item survey assessed baseline socioeconomic characteristics and putative COVID-19 exposures at home, work, social situations, and in the community. Baseline socioeconomic characteristics included preferred language at home, college education, occupation at the time of testing, access to employer-based insurance, and access to health care. Specific items included symptomatology at time of testing, mitigating behaviors (ie, mask usage, hand hygiene, and physical distancing [defined as the ability to maintain a distance over 6 feet]), participation in social gatherings with 10 or more people who were not all household members, use of public transportation, education, occupation, household characteristics, and receipt of safety-net financial benefits (unemployment benefits and stimulus checks under the CARES Act) in the 14 days before SARS-CoV-2 testing.^14^ Responses for most questions used either a 3-point Likert scale (never, sometimes, always) or were dichotomized as yes/no (eAppendix in the Supplement). This study followed the American Association for Public Opinion Research (AAPOR) reporting guideline.

### Statistical Analysis

The primary objective was to compare exposures and mitigating behaviors between participants testing positive and negative for SARS-CoV-2. The secondary objectives were to compare responses by Hispanic vs non-Hispanic ethnicity and to evaluate temporal trends in behaviors as an indicator of the effectiveness of public health messaging. For analysis of Likert scale responses, we dichotomized the responses as always or sometimes vs never.

We constructed logistic regression models to assess the association of SARS-CoV-2 test positivity with dichotomized survey responses for each response. To adjust for potential confounding by participant age and testing week, we retained these 2 variables in all models. We adjusted for sampling probability using survey weighting for each test week, as both factors may have influenced behaviors. The Mantel-Haenszel test of homogeneity was performed for data across racial and ethnic groups to determine whether a single adjusted odds ratio (aOR) for a variable could be presented. For the lone variable that revealed significant heterogeneity in its association with SARS-CoV-2 positivity (ie, mask wearing during breaks), separate logistic regression models for Hispanic and non-Hispanic participants were constructed. To compare differences in survey responses by ethnic groups, we calculated ORs with their respective 2-sided confidence intervals. Graphic visualizations were used to display temporal trends on selected behaviors, and we tested for statistical significance of the trends in mitigating exposures by racial and ethnic groups using the nonparametric test for trend. All statistical analyses were conducted using Stata software version 14.2 (StataCorp).

## Results

### Baseline Characteristics

During the 3 study weeks, 1682 adults were tested for SARS-CoV-2 at CCH facilities. Most tests were obtained from individuals who self-reported as Hispanic (740 [44.0%]) or Black (715 [42.5%]). The distribution of cases from each week of sampling was as follows: 250 (46%) on week 1, 250 (42%) on week 2, and 250 (21%) on week 3. Of 750 individuals randomly selected, 314 (41.9%) participated in the telephone survey (169 [53.8%] women). Of these, 159 (50.6%) participants self-reported as Hispanic and 155 (49.4%) self-reported as non-Hispanic, of whom 120 (77.4%) self-reported as Black, 23 (14.8%) as White, and 12 (7.7%) did not report or were unknown. Reasons for not participating in the study for 436 (58.1%) individuals included inability to reach after 3 phone call attempts, wrong phone number listed in the electronic health record, or disinterest in the survey.

Baseline characteristics of survey participants by SARS-CoV-2 status are shown in Table 1. Most participants who tested positive for SARS-CoV-2 were more likely to self-report as Hispanic (133 [63.6%]). Compared with survey participants who tested negative for SARS-CoV-2, participants who tested positive were more likely to be Spanish-dominant speaking (primary language is English: 75 of 209 [35.9%] vs 78 of 105 [74.3%]), work in factories or other industrial settings (49 [42%] vs 7 [17%]) or hospitality (16 [14%] vs 4 [9%]), have lower rates of college education (38 [18.2%] vs 35 of 105 [33.3%]) and employer-based medical insurance (32 [15.3%] vs 30 [28.6%]), have delayed testing for SARS-CoV-2 (median [interquartile range {IQR}] length of symptoms: 4 [4] days vs 2 [3.5] days), and to be more symptomatic at the time of SARS-CoV-2 testing (191 [91.4%] vs 46 [43.8%]).

**Table 1.** Baseline Characteristics of Survey Participants by SARS-CoV-2 Status

Characteristics	Participants, No. (%)	
	SARS-CoV-2 positive (n = 209)	SARS-CoV-2 negative (n = 105)
Gender		
Women	108 (51.6)	61 (58.1)
Men	101 (48.3)	44 (41.9)
Age, median (IQR), y	52 (42-61)	54 (44-62)
Race and ethnicity		
Hispanic	133 (63.6)	26 (24.8)
Non-Hispanic Black	57 (27.3)	63 (60.0)
Non-Hispanic other^a^	19 (9.1)	16 (15.2)
Primary language is English	75 (35.9)	78 (74.3)
College education	38 (18.2)	35 (33.3)
Symptomatic at time of testing	191 (91.4)	46 (43.8)
Length of symptoms, median (IQR), d	4 (3-7)	2 (1-4.5)
Occupation^b^		
Health care	17 (14)	10 (24)
Hospitality	16 (14)	4 (9)
Industrial	49 (42)	7 (17)
Retail	11 (9)	4 (9)
Transportation	9 (7)	5 (12)
Other	16 (14)	12 (29)
Occupation for household members^c^		
Agriculture	9 (7)	0
Healthcare	17 (14)	8 (18)
Hospitality	15 (13)	2 (4)
Industrial	40 (34)	6 (13)
Retail	24 (20)	15 (34)
Transportation	7 (6)	3 (7)
Other	7 (6)	10 (24)
Has medical insurance	32 (15.3)	30 (28.6)
Has primary care clinician	154 (73.7)	84 (80.0)
Test week		
Week 1	53 (25.4)	12 (11.4)
Week 2	102 (48.8)	31 (29.5)
Week 3	54 (25.8)	62 (59.0)

Abbreviation: IQR, interquartile range.

^a^ Non-Hispanic other includes non-Hispanic individuals who identified themselves as White (26 participants), Asian (2 participants), multiracial (2 participants), and other (5 participants).

^b^ Among participants who reported working outside the home in the 2 weeks prior to SARS-CoV-2 testing (160 participants total: 42 SARS-CoV-2 negative, 116 SARS-CoV-2 positive).

^c^ Among participants who reported family members who worked outside the home in the 2 weeks prior to SARS-CoV-2 testing (163 participants total: 44 SARS-CoV-2 negative, 119 SARS-CoV-2 positive).

### Factors Associated With SARS-CoV-2 Test Positivity

The association between testing positive for SARS-CoV-2 with self-reported exposure risks and mitigation practices for each response is shown in Table 2. Factors associated with a lower likelihood of testing positive among participants included reported mask use in any public setting (wore mask while running errands: aOR, 0.18; 95% CI, 0.07-0.42; at work: aOR, 0.23; 95% CI, 0.07-0.79; at social gatherings: aOR, 0.10; 95% CI, 0.00-0.50; in public transport: aOR, 0.00; 95% CI, 0.00-0.34) and hand sanitizer use (aOR, 0.26; 95% CI, 0.13-0.52). Factors associated with a higher likelihood of testing positive for SARS-CoV-2 included Hispanic ethnicity (aOR, 5.52; 95% CI, 4.30-7.08) and having a known COVID-19 contact at home (aOR, 15.18; 95% CI, 8.39-27.47) or at work (aOR, 4.66; 95% CI, 2.35-9.23).

**Table 2.** Patient Demographics and Transmission Mitigation Practices Associated With SARS-CoV-2 Status Among Survey Respondents

Characteristics	Participants, No. (%)		Odds of infection, aOR^a^ (95% CI)
	SARS-CoV-2 positive (n = 209)	SARS-CoV-2 negative (n = 105)	
Hispanic ethnicity	133 (63.6)	26 (24.8)	5.52 (4.30-7.08)
Exposure risks			
Worked outside the home	118 (56)	42 (40)	1.59 (0.82-3.10)
Household members worked outside the home	143 (68)	72 (69)	1.10 (0.52-2.32)
Attended social gatherings	51 (24)	18 (17)	1.53 (0.50-4.62)
Contact with COVID-19 cases			
At home^b^	94 (54)	5 (8)	15.18 (8.39-27.47)
At work^c^	70 (58)	11 (26)	4.66 (2.35-9.23)
At social gatherings^d^	17 (33)	2 (13)	3.35 (0.17-9.15)
Used public or shared transport	53 (25)	35 (34)	0.67 (0.24-1.86)
Household member used public or shared transport^e^	40 (24)	14 (25)	1.04 (0.67-1.60)
No. of household occupants, mean (SD)^f^	4.0 (1.9)	3.4 (1.8)	1.23 (0.84-1.92)
Mitigating behaviors			
Wore mask while running errands			
Always/sometimes	124 (59)	96 (91)	0.18 (0.07-0.42)
Never	85 (41)	9 (9)	
Wore mask while at work^c^			
Always/sometimes	73 (62)	38 (90)	0.23 (0.07-0.79)
Never	44 (38)	4 (10)	
Wore mask at work during breaks (non-Hispanic)^c^^,^^g^			
Always/sometimes	20 (59)	23 (74)	0.54 (0.09- 3.81)
Never	14 (41)	8 (26)	
Wore mask at work during breaks (Hispanic)^c^^,^^g^			
Always/sometimes	15 (91)	10 (91)	0.03 (0.00-0.46)
Never	69 (9)	1 (9)	
Wore mask on public transport^h^			
Always/sometimes	17 (32)	33 (94)	0.00 (0.00-0.34)
Never	36 (68)	2 (6)	
Wore mask at social gatherings^d^			
Always/sometimes	3 (6)	11 (79)	0.10 (0.00-0.50)
Never	44 (94)	3 (21)	
Wore gloves at work^c^			
Always/sometimes	78 (66)	30 (73)	0.80 (0.45-1.43)
Never	40 (34)	11 (27)	
Used hand sanitizer often	76 (36)	75 (71)	0.26 (0.13-0.52)
Washed hands often	157 (75)	90 (86)	0.55 (0.21-1.44)
Able to maintain physical distance at work^c^			
Always/sometimes	57 (48)	34 (81)	0.22 (0.02-1.70)
Never	61 (52)	8 (19)	
Able to maintain physical distance at work during breaks^c^			
Always/sometimes	75 (64)	41 (98)	0.04 (0-13.06)
Never	43 (36)	1 (2)	
Lives alone^i^	26 (13)	25 (25)	0.42 (0.16-1.08)
Safety net benefits			
Did not receive stimulus check	134 (64)	41 (40)	2.32 (2.12-2.54)
Did not receive unemployment benefits	183 (88)	85 (83)	1.32 (0.81-2.17)

Abbreviation: aOR, adjusted odds ratio.

^a^ aORs adjusted for age and test week.

^b^ Analysis performed only among participants who reported contact with COVID-19 cases at home (238 participants total: 63 SARS-CoV-2 negative, 175 SARS-CoV-2 positive).

^c^ Analysis performed only among participants who reported working outside the house (160 participants total: 42 SARS-CoV-2 negative [31 non-Hispanic, 11 Hispanic] participants, 118 SARS-CoV-2 positive [34 non-Hispanic, 84 Hispanic] participants).

^d^ Analysis performed only among participants who reported attending social gatherings (69 participants total: 18 SARS-CoV-2 negative, 51 SARS-CoV-2 positive).

^e^ Analysis performed only among participants who reported that a household member used public or shared transportation (221 participants total: 57 SARS-CoV-2 negative, 164 SARS-CoV-2 positive).

^f^ Analysis performed among participants who reported living with 1 or more persons (255 participants total: 178 SARS-CoV-2 positive, 77 SARS-CoV-2 negative).

^g^ Logistic regression stratified by ethnicity due to statistically significant interaction effect between the survey response and reported ethnicity.

^h^ Analysis performed only among participants who reported using public/shared transport (88 participants total: 35 SARS-CoV-2 negative, 53 SARS-CoV-2 positive).

^i^ Analysis performed among participants who reported stable housing (305 participants total: 99 SARS-CoV-2 negative, 209 SARS-CoV-2 positive).

### Exposure Risks, Mitigating Practices, and Safety-Net Benefits

Significant differences were noted in exposure risks, mitigating behaviors, and receipt of safety-net benefits across ethnic groups in the 2 weeks before SARS-CoV-2 testing (Table 3). Compared with non-Hispanic participants, Hispanic participants were more likely to work outside the home (aOR, 2.05; 95% CI, 1.27-3.30), participate in social gatherings (aOR, 2.15; 95% CI, 1.19-3.39), and report a known COVID-19 exposure at home. Hispanic participants were much less likely to have received a stimulus check from the CARES Act (aOR, 0.03; 95% CI, 0.02-0.07). There was no difference across ethnic groups in having a known COVID-19 exposure at work or at social gatherings. Additionally, there was no difference in use of public transportation by either participants or household members across groups.

**Table 3.** Differences in Exposure Risks, Mitigating Behaviors, and Safety-Net Benefits Between Participants by Ethnic Groups

Characteristics	Hispanic participants, No. (%) (n = 159)	Non-Hispanic participants, No. (%) (n = 155)	Odds ratio (95% CI)
Exposure risks			
Worked outside the home	95 (60)	65 (42)	2.05 (1.27-3.30)
Household members worked outside the home	116 (73)	99 (64)	1.52 (0.91-2.53)
Attended social gatherings	45 (28)	24 (15)	2.15 (1.19-3.39)
Contact with COVID-19 cases			
At home	80 (54)	19 (21)	4.52 (2.39-8.72)
At work^a^	53 (55)	28 (43)	1.59 (0.80-3.15)
At social gatherings^b^	14 (29)	5 (26)	1.51 (0.31-4.87)
Used public or shared transport	40 (25)	48 (31)	0.71 (0.40-1.58)
Household member used public or shared transport	33 (23)	21 (27)	0.80 (0.40-1.60)
No. of household occupants, mean (SD)	4.3 (1.9)	3.3 (1.7)	1.20 (0.81-1.92)
Mitigating behaviors			
Wore mask while running errands			
Always/sometimes	91 (57)	129 (83)	0.26 (0.15-0.46)
Never	68 (43)	26 (22)	
Wore mask while at work^a^			
Always/sometimes	58 (62)	53 (82)	0.36 (0.15-0.81)
Never	36 (38)	12 (18)	
Wore mask at work during breaks^a^			
Always/sometimes	25 (26)	43 (66)	0.18 (0.08-0.38)
Never	70 (74)	22 (34)	
Wore mask in public transport^c^			
Always/sometimes	11 (28)	39 (81)	0.08 (0.02-0.26)
Never	29 (72)	9 (19)	
Wore mask at social gatherings^b^			
Always/sometimes	2 (5)	12 (67)	0.02 (0.00-0.16)
Never	41 (95	6 (33)	
Wore gloves at work^a^			
Always/sometimes	61 (64)	47 (73)	0.64 (0.30-1.36)
Never	34 (36)	17 (27)	
Used hand sanitizer often	53 (33)	98 (63)	0.29 (0.17-0.47)
Washed hands often	123 (77)	124 (80)	0.85 (0.47-1.52)
Able to maintain physical distance at work^a^			
Always/sometimes	52 (55)	39 (60)	0.80 (0.40-1.60)
Never	43 (45)	26 (40)	
Able to maintain physical distance at work during breaks^a^			
Always/sometimes	60 (63)	56 (86)	0.27 (0.10-0.65)
Never	35 (37)	9 (14)	
Lives alone^d^	16 (10	35 (24)	0.34 (0.17-0.68)
Safety net benefits			
Did not receive stimulus check	139 (89)	36 (23)	0.03 (0.02-0.07)
Did not receive unemployment benefits	144 (92)	124 (80)	0.36 (0.16-0.74)

^a^ Analysis performed only among participants who reported working outside the house (total 160 participants: 95 Hispanic, 65 non-Hispanic).

^b^ Analysis performed only among participants who reported attending social gatherings (total 69 participants: 45 Hispanic, 24 non-Hispanic).

^c^ Analysis performed only among participants who reported using public/shared transport (total 88 participants: 40 Hispanic, 48 non-Hispanic).

^d^ Analysis performed among participants who reported stable housing (total 305 participants: 159 Hispanic, 146 non-Hispanic).

With regards to mitigation practices, rates of mask use in public settings (eg, running errands: aOR, 0.26; 95% CI, 0.15-0.46) and rates of hand sanitizer use (aOR, 0.29; 95% CI, 0.17-0.47) were significantly higher among non-Hispanic participants. There was no difference in reported hand washing or ability to maintain physical distance during work hours between groups. However, Hispanic participants reported less ability to maintain physical distance during work breaks (aOR, 0.27; 95% CI, 0.10-0.65). Lastly, non-Hispanic participants were more likely to have received a stimulus check (aOR, 0.03; 95% CI, 0.02-0.07) and unemployment benefits (aOR, 0.36; 95% CI, 0.16-0.74) compared with Hispanic participants in the 14 days before SARS-CoV-2 testing (Table 3).

 Statistically significant differences between Hispanic and non-Hispanic participants for exposure risks, mitigating practices, and safety-net benefits remained unchanged when only participants who tested positive for SARS-CoV-2 were included in the analysis.

### Temporal Trends in Behaviors

Four modifiable behaviors that could be influenced by public health messaging were evaluated for temporal trends across testing weeks (Figure). Overall, mask use increased across the 3 test weeks. There were statistically significant increases in the number of participants who reported wearing a mask while running errands (53.8% in week 1, 63.2% in week 2, 87.1% in week 3; *P* < .001), during work (51.2% in week 1, 69.2% in week 2, 86.3% in week 3; *P* = .001), or on public transport (31.8% in week 1, 37.9% in week 2, 86.5% in week 3; *P* < .001) across the 3 test weeks. Attending social gatherings appeared to increase during the second test week and then decrease. The percentage of Hispanic participants who reported consistent mask use in public settings remained lower across all 3 time points compared with non-Hispanic participants; attendance of social gatherings remained higher at all 3 time points. These differences remained unchanged when only participants who tested positive for SARS-CoV-2 were considered in the analysis, making recall bias less likely (data not shown).

**Figure.**
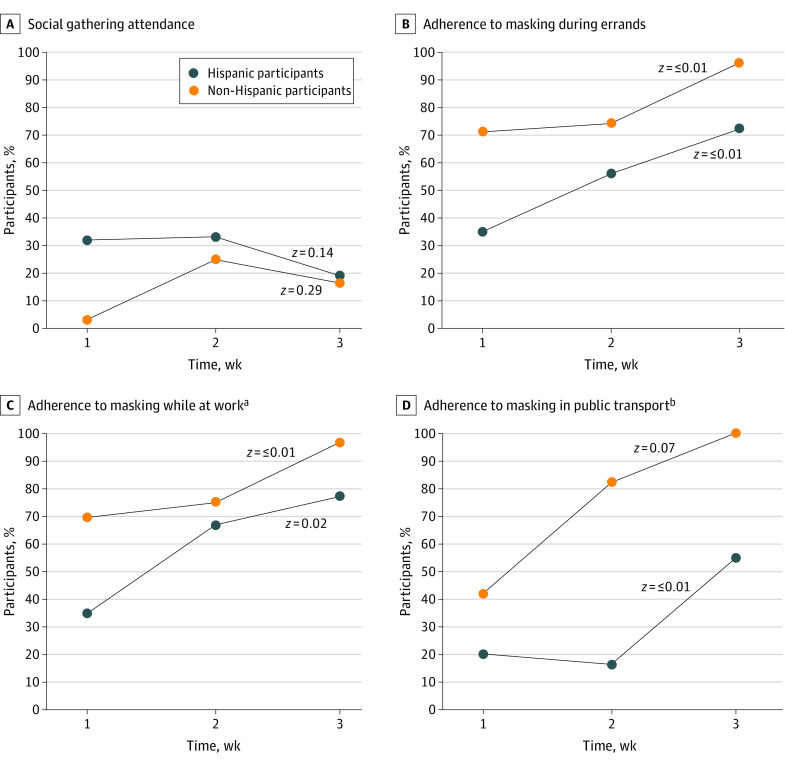
Trends in Social Gathering Attendance and Masking Across Survey Weeks by Ethnic Groups ^a^Analysis performed only among participants who reported working outside the house (160 participants in total: 95 Hispanic and 65 non-Hispanic). ^b^Analysis performed only among participants who reported using public or shared transportation (88 participants in total: 40 Hispanic, 48 non-Hispanic).

## Discussion

In this survey study of adults in a large US city, we found that the likelihood of SARS-CoV-2 positivity was associated with having known household or work contact exposures and Hispanic ethnicity. Individual adherence to public health messaging directing consistent mask and hand sanitizer use appeared protective against infection. When comparing exposure risks and mitigating practices among racial and ethnic groups, Hispanic participants were more likely to delay implementing preventive behaviors including mask use, physically distancing, and hand hygiene compared with non-Hispanic participants during each sampled week. Only 1 in 10 Hispanic participants reported receiving economic support through a CARES Act stimulus check compared with 3 out of every 4 non-Hispanic participants.

To our knowledge, this is the first study that has evaluated receipt of a stimulus check across Hispanic and non-Hispanic communities. Our finding that it was uncommon for Hispanic participants to receive financial support, which might have contributed to continued workplace exposures, is important and deserves further scrutiny. Eligibility for stimulus checks included US citizens who met income thresholds; however, non-US citizens and mixed immigration status individuals were not eligible. Additionally, individuals who met income requirements but did not file taxes in 2018-2019 or did not use direct deposit for past tax refunds may have experienced a delay in receiving the stimulus check.^14^ In this study, Hispanic participants were significantly less likely to have received a stimulus check compared with non-Hispanic participants. Immigration status was not collected in this survey, but could be inferred from census tract demographics and preference for the Spanish language, and may explain the lower accessibility to economic aid for many participants.^9^ Financial aid might offer protection from SARS-CoV-2 transmission in the workplace, as immigrant populations often work in overcrowded industries that do not offer job stability, employment protections, or sick time.^20,21,22^ Larger epidemiological studies are needed to corroborate our findings, but if economic aid offers a modicum of protection against SARS-CoV-2 infection, income-based universal aid may merit consideration as an additional tool in the fight against COVID-19. The American Rescue Plan Act of 2021 as a follow-up to the CARES Act provides an opportunity to assess this in more detail.^23^

As reported in previous studies, we found an increased risk of SARS-CoV-2 transmission from known household and work contacts, attributed to high-density living environments, shared workspaces, and prolonged workplace contact.^21,22,24^ We found that common community exposures such as social gatherings, use of public transportation, and work outside of the home did not increase the likelihood of SARS-CoV-2 positivity, apart from on-site dining, which was not assessed in this study but reported by Fischer et al.^25^ Importantly, and of value for public messaging, we corroborated that for our population consistent mask use was associated with a lower likelihood of test positivity.^9^ A novel finding in our study is the protective effect of hand sanitizers, suggesting their use may serve as a marker for enhanced SARS-CoV-2 preventive behaviors.

Interestingly, we found considerable differences in SARS-CoV-2 prevention measures between racial and ethnic groups. Hispanic participants reported significantly lower rates of mask use during work and on work breaks, on public transportation, while running errands, and at social gatherings. Possible barriers to mask use have been proposed, including mask availability, financial constraints, confusion and misinformation, poor penetration of public health messaging to non-English speaking populations, lower perceived susceptibility to COVID-19, physical and social discomfort, and perceptions of identity and autonomy.^26^ Further studies are needed to explore the barriers to wearing a face mask in the Hispanic population, which could inform public health measures and messaging. Additionally, Hispanic respondents reported more participation in social gatherings compared with non-Hispanic respondents, possibly from a lower perceived risk of COVID-19 among family and friends.^27^ The cultural tendency to gather in groups may partially explain the discrepancy in not maintaining physical distancing during work breaks as during work hours, in addition to potentially small workplace environments. Indeed, close family units, multi-generational households, and social cohesion are characteristics associated with the Hispanic culture and are often credited with countering health inequities.^28,29^ Lastly, this study demonstrated significant improvements in mask adherence and a curtailment in social gatherings over a 6-week time period in both Hispanic and non-Hispanic groups, although Hispanic groups delayed implementing mitigation strategies at all 3 time points. This underscores the need for more intensive and repeated messaging and community outreach to improve preventive behaviors in the Hispanic community.

### Limitations

This study has several limitations. First, the survey was conducted on a relatively small sample size, and those who declined to participate may have different exposure risks and behaviors not measured. Additionally, the small sample size may not be sufficiently powered to show measurable differences between racial and/or ethnic groups. Second, although participant awareness of results at the time of the survey may have influenced responses, our findings stratified by race and ethnicity remained statistically significant when restricted to those who tested positive for SARS-CoV-2. Third, surveys were conducted up to 8 weeks after SARS-CoV-2 testing was obtained, which may have amplified recall bias. Fourth, social desirability response bias may have overestimated self-reported adherence to preventive behaviors. Fifth, reliance on electronic health record–derived demographic data may have resulted in unforeseen misclassifications of race, ethnicity, and language preference.^30^ Sixth, as Hispanic participants were overrepresented among cases, this may have confounded the strength of our findings. Nonetheless, this study identified behaviors which likely can be modified through public health interventions including workplace modifications.

## Conclusion

Substantial changes in public policy are urgently needed while this pandemic still roils in order to address the social, economic, and health care disparities driving COVID-19 in Black and Hispanic communities. Based on the results of this survey study, public health messaging that is culturally adapted for and resonates with vulnerable populations, particularly Hispanic communities, must stress consistent mask use, enhanced hand hygiene, and physical distancing to stem the spread of SARS-CoV-2 and its variants while mass vaccination programs are rolled out. Larger studies are needed to evaluate the association of economic aid packages on SARS-CoV-2 transmission dynamics for all residents who meet income thresholds as a means to ease the burden of the pandemic and also lessen the risk for the general public.

## Notes

Supplement. **eAppendix.** Telephone Survey
