# Supplementary material for: Disparities and Temporal Trends in COVID-19 Exposures and Mitigating Behaviors Among Black and Hispanic Adults in an Urban Setting
Source: JAMA Netw Open. 2021 Sep 28;4(9):e2125187. doi: 10.1001/jamanetworkopen.2021.25187 (PMC8479580; doi:10.1001/jamanetworkopen.2021.25187)
Supplement: Supplement. — eAppendix. Telephone Survey [file jamanetwopen-e2125187-s001.pdf]

Supplemental Online Content

Badri S, Sardá V, Moncada JS, et al. Disparities and temporal trends in COVID-19 exposures and mitigating behaviors among Black and Hispanic adults in an urban setting. *JAMA Netw Open.* 2021;4(9):e2125187. doi:10.1001/jamanetworkopen.2021.25187

eAppendix. Telephone Survey

This supplemental material has been provided by the authors to give readers additional information about their work.

© 2021 Badri S et al. *JAMA Network Open.*

eAppendix. Telephone Survey

I understand that you were tested for COVID-19 several weeks ago. I'd like to ask you about your daily routine 2 weeks BEFORE you were tested for coronavirus, not what you are doing today. I understand people have changed their habits now, but we are interested in your day-to-day activities in the 2 weeks leading up to your COVID-19 test.

1. Were you infected with coronavirus? Yes No
   a. If yes, do you know how you were infected?

      Contact at home Contact at work Contact outside of work/home Don't know

2. Why did you get tested for coronavirus?
   a. I felt sick
   b. I was not sick, but I was exposed to someone
   c. I was not sick but went on my own to get tested
   d. I was not sick but my doctor ordered the test

3. If you were feeling sick, how many days were you sick before you were tested for coronavirus? ____ days

4. In the 2 weeks before you were tested for coronavirus, were you mainly at home? Yes No

5. Thinking of the time before you were tested for coronavirus, did you wear a mask outside the house when running errands, for example, going to the supermarket or bank?

   Never sometimes always

6. At the time you were tested for coronavirus, not now, were you working? IF NOT WORKING, SKIP TO QUESTION 15

   Not working working from home work outside the home.

7. If working outside the home, what type of work did you do?

   Retail (store); Agriculture (landscaping, farming); Health sector; Industrial/manufacturing sector; Hospitality (restaurant, hotel); Technology sector; transportation sector; education sector; government worker; other

8. If working outside the home, at the time you were tested for coronavirus, not now, were you able to stay 6 feet away from someone else during work? Never Sometimes Always
9. If working outside the home, at the time you were tested for coronavirus, not now, were you able to stay 6 feet away from someone else during breaks, for example eating lunch?
   Never Sometimes Always

10. If working outside the home, at the time you were tested for coronavirus, not now, were you using gloves during work? Never Sometimes Always

11. If working outside the home, at the time you were tested for coronavirus, not now, were you using a mask during work? Never Sometimes Always

    a. If never, why not?
       i. Did you feel unsafe wearing a mask in the community?
       ii. Were you unable to afford a mask?
       iii. Were you unable to find mask?
       iv. Personal choice/Felt uncomfortable

© 2021 Badri S et al. *JAMA Network Open.*

12. If working outside the home, at the time you were tested for coronavirus, not now, were you wearing a mask during breaks? Never Sometimes Always

    a. If never, why not?
        i. Did you feel unsafe wearing a mask in the community?
        ii. Were you unable to afford a mask?
        iii. Were you unable to find mask?
        iv. Personal choice/Felt uncomfortable

13. Who provided the mask at work? Employer Brought mask from home

14. If working outside the home, do you know if anyone at work was diagnosed with COVID-19 around the time you were tested? Yes No

    a. If yes, how many? (if yes, NUMBER only; if no, leave blank) _____________

15. Thinking of the 2 weeks before you were tested for coronavirus, did you use public transportation to get around the city? Yes No If No, SKIP to Question 16.

    a. If yes, what type of public transportation did you take?

       "L"/ bus / Metra Rideshare (Uber/Lyft) Carpool

    b. Did you wear a mask while taking public transportation? Never Sometimes Always

        1. If never, why not?
            a. Did you feel unsafe wearing a mask in the community?
            b. Were you unable to afford a mask?
            c. Were you unable to find mask?
            d. Personal choice/Felt uncomfortable

16. Before you were tested for coronavirus, not now, were you using hand sanitizer? Yes No

17. At the time you were tested for coronavirus, not now, were you washing your hands more frequently because of concerns of coronavirus? Yes No

18. At the time you were tested for coronavirus, did you have stable housing? Yes No
    If NO, GO TO QUESTION 26 after answering 18a.

    a. If no, where were you living? Family/friend's house shelter/street Rehab other

19. At the time you were tested for coronavirus, were you living alone? Yes No
    If YES, SKIP TO QUESTION 25

    a. If no, how many people were living at home with you (including you)? ____

20. How many bedrooms are in your home?

© 2021 Badri S et al. *JAMA Network Open.*

21. At the time you were tested for coronavirus, were any family members or housemates working outside the home? Yes No If No, SKIP TO QUESTION 25.

22. If yes, what type of work did family members/roommates do outside of the house?

    Retail (store); Agriculture (landscaping, farming); Health sector; Factory/manufacturing sector; Hospitality (restaurant, hotel); Technology sector; transportation sector; education sector; government worker; other

23. If living with others at time of coronavirus testing, did family members/housemates use public transportation, Uber/Lyft, or carpools ? Yes No

24. If living with others, was anyone in your household diagnosed with COVID-19 before or at the time you were tested? Yes No

    a. If yes, how many? (if yes, NUMBER only; if no, leave blank) _____________

25. What is the primary language spoken at home? English Spanish Other

26. Thinking of the time you were tested for coronavirus, did you participate in a social gathering outside of your home in the 2 weeks before your test? Yes No (If not, skip to Question 27)

    a. If yes, what type of social gathering do you go to? Select all that apply.

       Church service Party Family gathering Other

    b. Do you know if anyone at these gatherings was diagnosed with COVID-19? Yes No

    c. Did you wear a mask at social gatherings? Never Sometimes Always

       1. If never, why not?
          a. Did you feel unsafe wearing a mask in the community?
          b. Were you unable to afford a mask?
          c. Were you unable to find mask?
          d. Personal choice/Felt uncomfortable

27. Do you or anyone you live with work in the health care industry? Yes no
    a. If yes, where? Hospital Nursing Home Clinic Other

28. Have you or anyone you live with had any contact with someone who works in or was released from jail in the past month? Yes No

29. Have you or anyone you live with had any contact with someone who is homeless? Yes No

30. On May 5th, Governor Pritzker modified the stay at home orders and recommended everyone to wear a mask if unable to maintain social distancing (defined as maintaining a distance of 6 feet) from others.
    a. After May 5th, did you leave the house more? Yes no
    b. Did you were a mask? Never Sometimes Always
       i. If never, why not?
          1. Do you feel unsafe wearing a mask in the community?
          2. Were you unable to afford a mask?
          3. Were you unable to find a mask?

© 2021 Badri S et al. *JAMA Network Open.*

4. Personal choice/Felt uncomfortable

31. Did you receive a stimulus check? Yes no

32. Were you able to collect unemployment benefits? Yes no
    a. If yes, did this allow you to stay at home more easily? Yes no
    b. If no, did you continue to work on the side? Yes no

33. If you were unable to receive unemployment benefits, did you continue to work? Yes no

34. Do you have a regular doctor you see? Yes no

35. Do you have medical insurance through your employer? Yes no
    a. If no, were you avoiding going to the hospital for fear of medical expenses? Yes no

36. What grade level did you complete in school
    Elementary School High School College Graduate School

© 2021 Badri S et al. *JAMA Network Open.*
